# Association Between Carotid Artery Perivascular Fat Density and Cerebrovascular Ischemic Events

**DOI:** 10.1161/JAHA.118.010383

**Published:** 2018-12-07

**Authors:** Hediyeh Baradaran, Pavan K. Myneni, Praneil Patel, Gulce Askin, Gino Gialdini, Khalid Al‐Dasuqi, Hooman Kamel, Ajay Gupta

**Affiliations:** ^1^ Department of Radiology Boston University School of Medicine Boston MA; ^2^ Department of Radiology Weill Cornell Medicine New York NY; ^3^ Department of Healthcare Policy and Research Weill Cornell Medicine New York NY; ^4^ Department of Neurology Weill Cornell Medicine New York NY; ^5^ Clinical and Translational Neuroscience Unit Feil Family Brain and Mind Research Institute Weill Cornell Medicine New York NY

**Keywords:** adipose tissue, carotid artery, computed tomography angiography, Computerized Tomography (CT), Cerebrovascular Disease/Stroke, Vascular Disease

## Abstract

**Background:**

Studies have shown that pericoronary artery inflammation can be accurately detected via increased attenuation on computed tomography. Our purpose was to evaluate the association between pericarotid inflammation, measured by density of carotid perivascular fat on computed tomography angiography, with stroke and transient ischemic attack.

**Methods and Results:**

We screened computed tomography angiography examinations for patients with unilateral internal carotid artery (ICA) stenosis ≥50% to 99%. A blinded neuroradiologist placed regions‐of‐interest in the pericarotid fat on the slice showing maximal stenosis. Two‐sample *t* tests were performed to assess *between‐subject* differences in mean Hounsfield Units in carotid perivascular fat between symptomatic and asymptomatic patients. Paired *t* tests were used to assess *within‐subject* differences in mean Hounsfield Units between stenotic versus nonstenotic ICAs in a given patient. We included 94 patients, including 42 symptomatic and 52 asymptomatic patients. In the *between‐subject* analysis of stenotic ICAs, we found symptomatic patients had higher mean pericarotid fat density compared with asymptomatic patients (−66.2±19.2 versus −77.1±20.4, *P*=0.009). When comparing nonstenotic ICAs, there was no significant difference between pericarotid fat density in symptomatic compared with asymptomatic patients (−81.0±13.3 versus −85.3±18.0: *P*=0.198). *Within‐subject* comparison showed statistically significant increased density in stenotic ICA versus nonstenotic ICA with mean Hounsfield Units difference of 11.1 (*P*<0.0001).

**Conclusions:**

We found increased density, a surrogate marker for perivascular inflammation, in the fat surrounding ICAs ipsilateral to stroke or transient ischemic attack compared with asymptomatic ICAs. Our findings suggest that inflammation associated with culprit carotid plaques extends beyond the vessel lumen and can be identified using simple methods on computed tomography angiography imaging.

**Clinical Perspective**

**What Is New?**

- The density of the adipose tissue surrounding internal carotid arteries (ICAs) can be measured via Hounsfield Units on routine computed tomography angiography imaging and appears to be a marker for pericarotid fat inflammation.
- Using perivascular Hounsfield Units as measured on computed tomography angiography, there is significantly higher pericarotid fat density around ICAs ipsilateral to stroke or transient ischemic attack compared with ICAs, which are asymptomatic.
- Within a given patient, there are significantly higher Hounsfield Units around stenotic ICAs compared with nonstenotic ICAs.

**What Are the Clinical Implications?**

- Inflammation associated with symptomatic or “culprit” plaques appear to extend beyond the vessel lumen and can be identified and quantified on routine computed tomography angiography imaging.

## Introduction

Inflammation is central to the development of atherosclerosis.^1^ Serum markers of inflammation, such as plasma C‐reactive protein, can aid in the prediction of patients at high risk for cardiovascular disease,^2^, ^3^, ^4^ but are unable to pinpoint specific regions of vascular inflammation. Identification of localized perivascular inflammation would potentially be helpful in detecting patients who may benefit from targeted therapies aimed at the prevention of cardiovascular or cerebrovascular ischemic events.

Inflammatory changes in the fat surrounding the coronary arteries have been associated with coronary artery disease and high‐risk, rupture‐prone “culprit” plaques.^5^, ^6^ Recently, such changes have been shown to be detectable on computed tomographic (CT) imaging^6^, ^7^ as areas of increased perivascular fat density. Increased density of perivascular fat has been shown to closely correlate with histopathologic markers of inflammation, including increased pro‐inflammatory cytokines and macrophage activation.^8^ Despite several promising studies using CT in the assessment of pericoronary artery fat inflammation, the value of carotid artery perivascular fat density measurements remains uncertain. Our aim was to evaluate the association of pericarotid fat inflammation, as measured by the attenuation in the carotid perivascular adipose tissue on CT angiography (CTA), with cerebrovascular ischemic symptoms.

## Methods

The data that support the findings of this study are available from the corresponding author upon reasonable request.

We screened consecutive neck CTA examinations performed at our institution from January 1, 2014 through December 31, 2015 to identify patients meeting the following inclusion criteria: (1) unilateral extracranial internal carotid artery (ICA) stenosis between 50% and 99% according to NASCET (North American Symptomatic Carotid Endarterectomy Trial)^9^ criteria identified on the CTA neck examination; (2) adequate information within the medical record to ascertain history of ischemic stroke or transient ischemic attack (TIA); (3) adequate information within the medical record regarding pre‐existing vascular risk factors. To minimize the risk of including patients in whom a cardioembolic stroke mechanism was present, we excluded any patients who had simultaneous bilateral anterior circulation events. Patients with bilateral extracranial ICA stenosis were also excluded. This study was approved by the Institutional Review Board at our institution. The need for informed patient consent was waived by the Institutional Review Board.

### Imaging Technique

CTA neck studies were performed on one of our institution's CT scanners, including the Optima 660, Lightspeed Xtra, Lightspeed Pro‐16 or Discovery HD‐750 (GE Healthcare; Milwaukee, WI). CTA studies were acquired in helical scanning mode with coverage extending from the aortic arch to the C1 ring. Studies were collimated at 0.625 mm, with kVp of 120, auto‐mA, and with a rotation time of 0.5 s. For each study, ≈90 mL of nonionic iodinated contrast (iohexol, Omnipaque, GE Healthcare) was administered at a rate of 4 to 5 mL/s using a power injector and SmartPrep region‐of‐interest on the aortic arch via an 18‐gauge peripheral intravenous catheter.

### Imaging Data Analysis

Degree of stenosis was determined using the NASCET method^9^ by a radiologist blinded to clinical data for the stenotic ICA. As per NASCET trial guidelines, in cases of near occlusion (ie, string sign), a 95% stenosis value was assigned to the ICA.

Evaluation of the density of the perivascular fat surrounding the carotid artery was performed by a neuroradiologist blinded to clinical data, with densities measured in Hounsfield Units (HU). Using predefined image display settings (window width, 500 HU; window center, 100 HU), pixels corresponding to adipose tissue were identified. We adapted an established approach used in the coronary arteries,^6^ and placed 2 regions‐of‐interest (ROI) (each 2.5 mm^2^) in the perivascular fat present on the same axial slice showing the maximal NASCET‐defined ICA stenosis (Figure). The site of ROI placement was not exactly the same for each subject and was determined by location of the site of maximum stenosis, location of the carotid plaque, and location of the perivascular fat pads. Mean and maximum HU values were obtained from the ROIs. ROIs were drawn carefully to only include detectable fat density (visually dark and confirmed by negative HU). Care was taken to exclude the carotid artery wall or surrounding soft‐tissue structures, with ROIs placed at least 1 mm from the outer margin of the carotid artery wall. If no detectable stenosis was present in a given ICA contralateral to an index ICA with ≥50% stenosis, ROIs were placed in the fat surrounding the ICA on the same axial slice as the contralateral ICA demonstrating maximum stenosis. To test for the reliability of the measurements, a second neuroradiologist blinded to the clinical data and the initial measurements repeated ROI measurements on a subset of 40 arteries.

**Figure 1:**
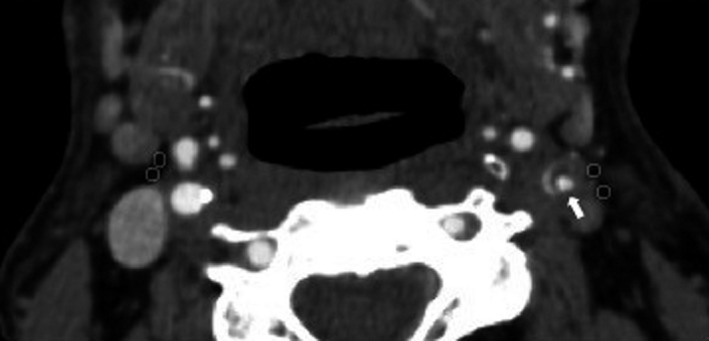
Computed tomography angiography image of a 72‐year‐old man presenting with a left middle cerebral artery territory infarction. Two regions‐of‐interest (ROI) were placed in the perivascular fat surrounding the stenotic (arrow) left internal cerebral artery (ICA) and 2 ROIs were placed in the perivascular fat surrounding the nonstenotic right ICA on the same axial slice. In this case, the 2 left ROIs were −65.3 and −62.8 and the 2 right ROIs were −80.3 and −78.9.

### Clinical Data Analysis

History of any prior stroke or TIA was determined by a neurologist blinded to the imaging data after reviewing the electronic medical records. Ischemic stroke was defined as permanent neurological dysfunction caused by focal brain or retinal ischemia. TIA was defined as a transient episode of neurological dysfunction caused by brain or retinal ischemia.^10^ Only those ischemic events occurring in the vascular territory supplied by the index ICA were considered symptomatic. Vascular risk factors for each patient were also collected including history of diabetes mellitus (hemoglobin A1c >6.5% or on diabetic medications), hypertension (blood pressure >140/90), history of smoking, atrial fibrillation, hyperlipidemia (low‐density lipoprotein >100 or on statin), and coronary artery disease.

### Statistical Analysis

We performed both *between‐subjects* and *within‐subjects* analyses. First, we evaluated whether there is greater *localized* perivascular inflammation near a symptomatic ICA versus an asymptomatic ICA. We used a 2‐sample *t* test to perform a between‐subject comparison of perivascular fat density around the stenotic ICA for patients who were symptomatic compared with those who were asymptomatic. We assessed differences in both mean and maximum density measurements. Second, we evaluated whether there is greater *systemic* perivascular inflammation in patients experiencing ischemic events. For this we used a 2‐sample test to perform another between‐subject comparison of the perivascular fat density near the contralateral, nonstenotic ICAs in patients who were symptomatic compared with fat near the nonstenotic ICAs in asymptomatic patients. Third, we compared the perivascular fat density *within‐subjects*, comparing the stenotic ICA to the contralateral ICA without significant stenosis. Paired *t* tests were used to assess the mean and maximum difference in perivascular fat densities between left and right sides of the same patient. For the sensitivity analyses, we used the Wilcoxon rank‐sum test for non‐normal data. We calculated the correlation of degree of NASCET stenosis in each ICA with the mean and maximum HU using the Spearman correlation coefficient for non‐normal data. We calculated an intraclass correlation coefficient and corresponding 95% CI to evaluate the degree of inter‐reader reliability. All fat density measurements are reported in HU.

## Results

### Cohort Characteristics

We screened 1307 neck CTA examinations. Out of 230 patients with ≥50% carotid stenosis, we excluded 55 patients for having bilateral carotid artery stenosis >50%, 45 patients for having carotid artery occlusion, and 2 patients for technical limitations precluding accurate assessment of the pericarotid fat. Out of the 94 patients included, the average age was 73.4 years and 38.3% were female. A total of 52 patients (55.3%) were asymptomatic, while 42 (44.7%) were symptomatic (Table 1). Of the symptomatic patients, 29 patients had strokes and 13 had TIAs. The overall mean HU of the pericarotid fat was −72.3±20.5 and the overall maximum HU was −59.4±22.5.

**Table 1:** Patient Demographics

Characteristic	Overall (n=94)	Symptomatic (n=42)	Asymptomatic (n=52)	*P* Value
Age (y), SD	73.4 (9.9)	72.4 (11.2)	74.2 (8.8)	0.397
Female	36 (38.3%)	16 (38.1%)	20 (38.5%)	
Hypertension	71 (75.5%)	30 (71.4%)	41 (78.8%)	0.554
Diabetes mellitus	24 (25.5%)	7 (16.7%)	17 (32.7%)	0.125
Hyperlipidemia	55 (58.5%)	27 (64.3%)	28 (53.8%)	0.417
Atrial fibrillation	18 (19.1%)	8 (19.0%)	10 (19.2%)	0.433
Coronary artery disease	33 (35.1%)	15 (35.7%)	18 (34.6%)	0.412
Smoking history	54 (57.4%)	22 (52.4%)	32 (61.5%)	0.456
Heart failure	14 (14.9%)	6 (14.3%)	8 (15.4%)	0.474
COPD	10 (10.6%)	6 (14.3%)	4 (7.69%)	0.334
Chronic kidney disease	6 (6.38%)	4 (9.52%)	2 (3.85%)	0.402
Antithrombotic therapy	73 (77.7%)	30 (71.4%)	43 (82.7%)	0.292
Aspirin	52 (55.3%)	20 (47.6%)	32 (61.5%)	0.254
Clopidogrel	27 (28.7%)	11 (26.2%)	16 (30.8%)	0.796
Warfarin or NOAC	14 (14.9%)	5 (11.9%)	9 (17.3%)	0.660
Other antithrombotics	4 (4.26%)	3 (7.14%)	1 (1.92%)	0.321
Statins	63 (67.0%)	25 (59.5%)	38 (73.1%)	0.242
Antihypertensive therapy	71 (75.5%)	30 (71.4%)	41 (78.8%)	0.555
β‐Blockers	46 (48.9%)	20 (47.6%)	26 (50.0%)	0.982
Calcium antagonists	21 (22.3%)	14 (33.3%)	7 (13.5%)	0.403
Diuretics	28 (29.8%)	11 (26.2%)	17 (32.7%)	0.647
ACE inhibitor	22 (23.4%)	10 (23.8%)	12 (23.1%)	0.803
AT‐1 antagonist	18 (19.1%)	7 (16.7%)	11 (21.2%)	0.775
Other antihypertensive medications	4 (4.26%)	1 (2.38%)	3 (5.77%)	0.626
Antidiabetic therapy	22 (23.4%)	6 (14.3%)	16 (30.8%)	0.103
Insulin	7 (7.45%)	1 (2.38%)	6 (11.5%)	0.126
Oral hypoglycemic or antidiabetic drugs	19 (20.2%)	5 (11.9%)	14 (26.9%)	0.123

The 2‐sample *t* test was used to assess the relationship between symptom status and age. The χ^2^ test or Fisher exact test, as appropriate based on cell size, was used to assess the relationship between symptom status and all discrete variables. ACE indicates angiotensin‐converting enzyme; AT‐1, antithrombin‐1 antagonists; COPD, chronic obstructive pulmonary disease; NOAC, novel oral anticoagulant.

### Comparison of ICAs Between Subjects

In the between‐subjects analysis of stenotic ICAs, we found that symptomatic patients had a significantly higher pericarotid fat density compared with asymptomatic patients (−66.2±19.2 versus −77.1±20.4 mean HU, *P*=0.009; −51.2±20.3 versus −66.2±19.2 maximum HU, *P*=0.001; Table 2). When comparing the fat density around the nonstenotic ICAs, there was no significant difference between the mean HU in symptomatic patients compared with asymptomatic patients (−81.0±13.3 versus −85.3±18.0: *P*=0.198) and a significant difference between the maximum HU between symptomatic and asymptomatic patients (−76.0±18.6 versus −68.7±16.0; *P*=0.049). In a sensitivity analysis of the symptomatic patients, there was no difference between fat density values between stroke and TIA patients (−69.7±17.4 versus −58.4±21.3; *P*=0.141 for mean HU; −53.2±19.8 versus −46.0±21.6; *P*=0.473 for max HU). In a sensitivity analysis limited to the 29 ischemic stroke patients, there was significantly higher maximum fat density around the stenotic ICA compared with the stenotic ICA of asymptomatic patients (−53.2±19.8 versus −65.7±22.2 HU; *P*=0.013). We found a very weak negative correlation between degree of NASCET stenosis in each ICA with the mean and maximum HU with *r*=−0.04 and *r*=−0.001, respectively.

**Table 2:** Pericarotid Fat Density (Measured in HU) on Computed Tomography Angiography

Characteristic	Results			*P* Value
	Overall (n=94)	Symptomatic (n=42)	Asymptomatic (n=52)	
Overall mean HU	−72.27 (20.5)	−66.23 (19.2)	−77.14 (20.4)	0.009
Overall max HU	−59.37 (22.5)	−51.24 (20.3)	−65.94 (22.2)	0.001
NASCET, % stenosis	67.6 (12.5)	67.9 (13.9)	67.4 (11.3)	0.873

The 2‐sample *t* test was used to assess the relationship between mean and maximum fat density (HU) and symptom status. Symptomatic patients have had a prior stroke or transient ischemic attack ipsilateral to the stenotic internal carotid artery. HU indicates Hounsfield Units; NASCET, North American Symptomatic Carotid Endarterectomy Trial.

### Comparison of ICAs Within Subjects

We also performed within‐subjects analysis comparing pericarotid fat density differences between the 2 ICAs (pericarotid fat density of the stenotic ICA minus the pericarotid fat density of the nonstenotic ICA). We found significantly increased pericarotid fat density around the stenotic ICA versus the nonstenotic ICA (mean and maximum HU differences of 11.1 and 13.4, respectively, both *P*<0.001) (Table S1). For the subgroup of symptomatic patients (n=42), we also found that the stenotic ICA pericarotid fat was significantly higher in density than the nonstenotic ICA pericarotid fat (mean and maximum HU difference of 14.7 and 17.5, respectively, both *P*<0.001). Similarly, for the subgroup of asymptomatic patients (n=52), we also found the stenotic ICA pericarotid fat density to be significantly higher than the nonstenotic ICA pericarotid fat (mean and maximum HU differences of 8.1 and 10.3, respectively; *P*<0.006 and *P*=0.001, respectively).

### Reproducibility of Fat Density Assessments

On a subset of 40 ICAs, we had good inter‐rater reliability with an interclass coefficient of 0.83 (0.63, 0.93) and 0.94 (0.87, 0.98) for mean HU and maximum HU, respectively (Table S2). The largest difference observed between the 2 readers on a single case was 22 HU.

## Discussion

In a cohort of patients with 50% to 99% unilateral carotid stenosis, we used clinically routine CTA studies to demonstrate increased density in the pericarotid fat surrounding the stenotic ICAs of patients who had a history of ipsilateral stroke or TIA compared with patients who were asymptomatic. Additionally, when comparing the 2 ICAs within a given patient, we found a significant difference in pericarotid fat density in stenotic ICAs compared with nonstenotic ICAs, a finding present in both symptomatic and asymptomatic patients. These findings were present with only a very weak correlation between degree of NASCET stenosis and pericarotid fat density. Given recent data supporting the role of perivascular density changes as a surrogate marker for localized inflammation,^8^ our findings overall suggest that there is increased inflammation in the fat surrounding symptomatic ICAs in patients with ischemic events secondary to carotid disease, and that this local inflammation is likely independent of any generalized systemic inflammation, given the relatively lower density of the nonstenotic ICA pericarotid fat. Our findings also suggest that the presence of significant carotid artery stenosis is associated with increased perivascular fat inflammation, regardless of the presence of ipsilateral neurologic symptoms. These findings are similar to emerging findings in the coronary artery literature suggesting that increased fat density surrounding coronary artery plaques on CT is associated with perivascular inflammation on histopathology and can be used to identify “culprit” plaques.^6^, ^8^

The relationship between the vascular wall and the surrounding adipose tissue is complex and likely bidirectional.^11^, ^12^ Many studies suggest that paracrine effects, rather than endocrine effects, dominate in the perivascular fat and it is hypothesized that the vascular wall releases proinflammatory cytokines that disrupt the differentiation of perivascular adipose tissue.^13^, ^14^ These inflammatory changes in perivascular fat can be assessed by differences in the fat attenuation as measured by HU on CT. While markers of systemic inflammation including serum C‐reactive protein and other inflammatory cytokines have been associated with increased risk of stroke^15^ and plaque instability,^16^ these systemic markers of inflammation are unable to pinpoint specific vascular beds or plaques as unstable or high risk. By evaluating the perivascular fat with a noninvasive imaging technique, local inflammation can be more accurately assessed, thereby potentially providing opportunities for more targeted treatment.

Though a developing body of literature now exists supporting CT‐based techniques to evaluate for high‐risk features in carotid plaque,^17^ the role of CT in evaluating for detectable changes in fat surrounding the vessel wall is uncertain. Our study's major finding—that there are significant differences in perivascular inflammation detectable in pericarotid adipose tissue—warrants future prospective studies of first‐time and recurrent stroke risk stratification using fat density measurements, as well as confirmatory histopathologic studies using carotid endarterectomy specimens. Adapting similar techniques from studies in the coronary arteries, we evaluated the density of pericarotid adipose tissue on CT by measuring HU, using standard imaging protocols from our institution in a technique that was highly reproducible. The robustness of this approach lends itself to future prospective study across institutions and different CT scanning hardware. While it is still uncertain if the perivascular inflammation is present as a result of plaque rupture after the plaque has become symptomatic, or if we can detect inflammation surrounding “unstable” plaques before they result in cerebrovascular ischemia, we have evidence that perivascular inflammation can be detected via a simple method using routine CTA neck examinations. Though we detected statistically significant differences between groups, with our modest sample size we were not able to produce reliable data regarding numeric cutoff points for discriminating between symptomatic and asymptomatic patients. Future work is necessary to determine how well these biomarkers can discriminate between patients in clinical practice.

Our study has some limitations. Since our study was cross‐sectional and retrospective in design, the ability of this technique to predict future stroke is uncertain. Future prospective studies evaluating the predictive ability of increased fat attenuation as a marker for pericarotid inflammation would be helpful to see whether this would be a viable stroke risk biomarker. Furthermore, the measurement of density on CT can be affected by variability in ROI placement. In order to ensure uniform ROI placement, we chose to draw ROIs at the site of greatest stenosis in the stenotic ICAs and drew ROIs at the same axial slice on the contralateral ICAs, even though these may not have been the sites with the greatest degree of inflammation. Despite this, our density measurements showed high reproducibility with 2 independent observers, suggesting that inter‐reader differences are likely to be relatively modest using this technique. Finally, our HU measurements were made on different CT scanners across different platforms and thus can affect the HU values,^18^ which may limit our ability to compare these values among patients. While this may make it difficult in the future to create a single threshold value to indicate inflammation, we still observed robust, statistically significant differences among patients who were scanned on different platforms. Furthermore, for many of our analyses, we used the patient's contralateral ICA as a control, which mitigates the issues of HU differences across CT scan techniques and platforms.

In summary, we found that the density of perivascular fat, a surrogate marker for perivascular fat inflammation, is significantly higher in ICAs ipsilateral to a stroke or TIA compared with those ICAs that are asymptomatic. We also found increased fat density adjacent to stenotic but asymptomatic carotid arteries when compared with nonstenotic arteries. Our findings suggest that the inflammatory process associated with carotid atherosclerotic disease extends beyond the vessel lumen and can be quantified noninvasively using simple methods on routine CTA imaging.

## Disclosures

None.

## Supporting information


**Table S1.** Within‐Subject Comparison
**Table S2.** Inter‐Reader Variability for Mean and Maximum Hounsfield Units (HU) on Computed Tomographic Angiography
