# Supplementary material for: Association Between Carotid Artery Perivascular Fat Density and Cerebrovascular Ischemic Events
Source: J Am Heart Assoc. 2018 Dec 7;7(24):e010383. doi: 10.1161/JAHA.118.010383 (PMC6405622; doi:10.1161/JAHA.118.010383)
Supplement: Supplementary file 1 — Table S1. Within‐Subject Comparison Table S2. Inter‐Reader Variability for Mean and Maximum Hounsfield Units (HU) on Computed Tomographic Angiography [file JAH3-7-e010383-s001.pdf]

SUPPLEMENTAL MATERIAL

Table S1. Within Subject Comparison.

	Results		
	**Mean HU difference**	**Max HU difference**	**p-value**
**All Patients**	11.1 (18.8)	13.4 (20.3)	<0.001
**Symptomatic Patients (n=42)**	14.7 (16.3)	17.5 (17.8)	<0.001
**Asymptomatic Patients (n=52)**	8.1 (20.3)	*	0.001

*We do not report mean difference for non-parametric tests.
The two-sample t-test was used to assess the relationship between mean and max fat density (HU) difference.

Difference in pericarotid fat density (measured in Hounsfield Units) on computed tomography angiography between the stenotic and nonstenotic internal carotid arteries. Stenosis means >70% by NASCET criteria. Symptomatic patients have had a prior stroke or TIA ipsilateral to the stenotic ICA. HU=Hounsfield Unit; NASCET=North America Symptomatic Carotid Endarterectomy Trial. ICA= internal carotid artery.

Table S2. Inter-reader variability for mean and maximum Hounsfield Units (HU) on computed tomographic angiography.

	**Inter-reader reliability**	
	**Mean HU**	**Max HU**
**ICC**	0.83 (0.63, 0.93)	0.94 (0.87, 0.98)

ICC= interclass coefficient
